# Antibodies to Glycoproteins Shared by Human Peripheral Nerve and *Campylobacter jejuni* in Patients with Multifocal Motor Neuropathy

**DOI:** 10.1155/2013/728720

**Published:** 2013-05-13

**Authors:** Ljubica Suturkova, Katerina Brezovska, Ana Poceva-Panovska, Aleksandra Grozdanova, Sladjana Knežević Apostolski, Ivana Basta

**Affiliations:** ^1^Faculty of Pharmacy, Ss. Cyril and Methodius, University Skopje, Macedonia; ^2^Outpatient Neurological Clinic, Belgrade, Serbia; ^3^Neurological Clinic, Clinical Centre of Serbia, Belgrade, Serbia

## Abstract

We have tested serum samples from 24 patients with multifocal motor neuropathy (MMN) for reactivity to ganglioside GM1 and to Gal(**β**1–3)GalNAc-bearing glycoproteins isolated from human peripheral nerve and from *Campylobacter jejuni* (*Cj*) serotype O:19. IgM anti-GM1 antibodies were detected by ELISA in 11 patients (45.8%) with MMN and in only one subject (4%) from the control group. Western blots showed positive reactivity of sera from 6 patients (25%) with MMN to several Gal(**β**1–3)GalNAc-bearing glycoproteins from human peripheral nerve and from *Cj* O:19 isolates. Sera from three patients (12.5%) with MMN showed positively reactive bands with similar electrophoretic mobility in all isolates (60–62 kDa, 48–51 kDa, 42 kDa, and 38 kDa). All six patients showed positive reactivity to 48–52 kDa protein isolated from human peripheral nerve. Increased titer of IgG antibodies to 60–62 kDa protein isolated from *Cj* O:19 associated with Guillain-Barré syndrome was detected in three patients, and their serum showed also IgG positive reactivity to peripheral nerve antigen with the same electrophoretic mobility. One of these patients had a previous history of *Cj* infection which suggests the possibility that *Cj* may be also involved in the pathogenesis of MMN.

## 1. Introduction

Multifocal motor neuropathy (MMN) is a chronic immune mediated neuropathy characterized by asymmetric, predominantly distal upper limb weakness, no sensory impairment, and by the presence of multifocal persistent partial conduction blocks on motor but not sensory nerves [1]. The muscle weakness related to individual motor nerve is associated with motor conduction block, at site distinct from common entrapment or compression syndromes [2]. Serum IgM antibodies to ganglioside GM1 were reported in 22–85% of patients with MMN, and these striking differences in reported prevalences may be explained by different laboratory techniques [3]. IgM antibodies against other gangliosides than GM1 have also been reported in MMN. Antecedent *Campylobacter jejuni *(*Cj*) infection has been implicated in the induction of Guillain-Barré syndrome (GBS) by a mechanism of molecular mimicry with the lipopolysaclharides (LPS) of *Cj *[4]. The pure motor axonal GBS is associated with antibodies to gangliosides GM1, GD1a, GalNAc-GD1a, GD1b, and GM1b [5–7]. We have previously shown the reactivity of anti-GM1 and asialo-GM1 antibodies from patients with MMN or chronic neuropathies with the LPS of *Cj *[8]. The possibility that *Cj* may also be involved in the pathogenesis of MMN has been supported by several reports of patients developing MMN and high titers of anti-GM1 antibodies after *Cj* enteritis [9–12]. 

The Penner's O:19 serotype of *Cj* contains LPS with GM1-like oligosaccharidesm determinants and is most commonly associated with pure motor GBS [5, 13, 14]. Cross-reactive determinants were detected in glycoproteins from human peripheral nerve and *Cj* O:19, recognized by peanut agglutinin (PNA) and by GM1 positive sera from patient with GBS associated with *Cj* infection [15, 16]. The aim of this study was to investigate the cross-reactivity of GM1 positive sera from patients with MMN and GM1-like protein antigens isolated from human peripheral nerve and from *Cj* O:19.

## 2. Material and Methods

### 2.1. Serum Samples

Serum samples from twenty-four patients with MMN diagnosed at the Neurological Clinic of the Clinical Center of Serbia and at the Outpatient Neurological Clinic were used in the study. As a positive control, sera from patients with GBS following *Cj* infection were used. These patients had high titer of anti-GM1 antibodies cross-reactive to glycoproteins from human peripheral nerve and from *Cj* O:19. As a negative control, sera from five patients with other neurological diseases (motor neuron disease (MND), multifocal sensory motor neuropathy (MSMn)) and sera from 24 volunteer healthy subjects were used. 

### 2.2. Isolation of Glycoproteins from Human Peripheral Nerve

Human peripheral nerve was obtained at autopsy within 8 hr after death from patients who died from non neurological disease and was kept frozen at −70°C (Department of Forensic Medicine, Faculty of Medicine, Ss. Cyril and Methodius University, Skopje, Macedonia). Neural tissue was pulverized in liquid nitrogen, delipidated with chloroform : methanol (1 : 2) solution, solubilized by homogenization (MICROSON, ultrasonic cell disruptor XL, Misonix Incorporated, NY, USA) in 0.5% Triton X-100, 0.4% SDS with protease inhibitor cocktail, and heated at 65°C for 10 min. The insoluble matter was removed by centrifugation at 4200 rpm for 45 min at room temperature [17]. Protein isolates were lyophilized and kept on −70°C until use.

### 2.3. Isolation of Glycoproteins from *C. jejuni* (O:19)

Bacterial protein isolates were obtained from two strains of *Cj* serotype O:19. The first strain was commercial strain of *Cj* O:19, ATCC 700297, isolated from patient with pure motor axonal form of GBS from China (American Type Culture Collection (ATCC), Rockville, MD, USA). The second strain was strain of *Cj* O:19, isolated from patient with bacterial enteritis, *C. jejuni*-enteritis, which is not associated with development of GBS (Institute for Microbiology and Parasitology, Faculty of Medicine, Ss. Cyril and Methodius, University Skopje, Macedonia). *Cj* strains were cultured in Campylobacter agar (Campylosel, bioMérieux, France). The bacteria were grown at 42°C for 48 h under microaerophilic conditions (5% O_2_, 10% CO_2_, and 85% N_2_, CampyGen, Oxoid). The *Cj* was identified by confirming the morphological macro- and microscopic characteristics of the developed colonies, by determining the mobility, staining according to Gram and with suitable biochemical tests (oxidase, catalase, and hippurate hydrolysis) at the Institute for Microbiology and Parasitology, Faculty of Medicine, Skopje. The serotyope O:19 was confirmed using commercial kit for serotyping (Denka Seiken, Tokyo, Japan).

Further multiplication of the bacteria was performed on Columbia agar (Oxoid) at 42°C for 48 h under microaerophilic conditions (5% O_2_, 10% CO_2_, and 85% N_2_, CampyGen, Oxoid). Bacterial cells from 20 petri dishes for each strain were collected in 0,9% NaCl w/v and centrifuged at 4000 rpm for 30 min. Pellets were resuspended in 8.0 mL 0.1 M Tris-HCl (pH 7.8) and ultrasonically disrupted (MICROSON, ultrasonic cell disruptor XL, Misonix Incorporated, NY, USA). After centrifugation (45 min; 4200 rpm) proteins in the supernatant were dialyzed twice against 0.1 M Tris-HCl (pH 7.5) at 4°C for 3 h, lyophilized and kept on −70°C until use [18].

### 2.4. Purification of Gal-GalNAc-Bearing Glycoproteins

Gal-GalNAc-bearing glycoproteins from the human peripheral nerve and *Cj* (O:19) were purified by affinity chromatography, using agarose-bound Peanut agglutinin (PNA) (Sigma-Aldrich) as described by Apostolski et al. [17].

### 2.5. SDS-PAGE and Western Blot

Following separation on 10% acrylamide/bisacrylamide gel (20 *μ*g total glycoproteins per well) by sodium dodecyl sulphate-polyacrylamide gel electrophoresis (SDS-PAGE), purified glycoproteins were transferred electrophoretically onto nitrocellulose sheets. Unreactive binding sites were blocked in 8% BSA in Tris buffered saline (TBS-0.02 M Tris base, 0.5 M NaCl, pH 7.5), 1 hour at room temperature. The blots were washed three times with TBS containing 1% Tween 20 and incubated overnight with sera from patients diluted 1 : 100. After washing, the membranes were incubated with anti human IgG antibodies, conjugated with peroxidase, (Sigma-Aldrich, USA), diluted 1 : 1000, for 1 hour at room temperature. Visualization of the reaction products was done using diaminobenzidine (DAB), 15 mM imidazole, and 0.025% H_2_O_2_ in water (BioRad Laboratories, Hercules, CA, USA).

### 2.6. Purification of the Glycoproteins with Electrophoretic Mobility between 60 and 62 and between 48 and 50 kDa, by Preparative SDS-PAGE

Immunoreactive proteins with electrophoretic mobility between 60 and 62 and between 48 and 50 kDa were purified using preparative SDS-PAGE. Total protein extracts (approximately 40 *μ*g total glycoproteins/well) were electrophoretically separated using 10% acrylamid/bisacrylamide gel, using vertical electrophoresis system (Protean IIxi, BioRad). Separated proteins were visualized by immersing the gel in ice cold solution of 1 mM dithiothreitol (DTT) and 0.25 M KCl. On the black background proteins are seen as white bands. Protein standard with the molecular weights in the range of 7–212 kDa (Prestained SDS-PAGE standards, broad range, Bio-Rad) was used as a marker for the molecular weight. The bands of interest (mobility between 60 and 62 and between 48 and 50 kDa) were cut on small pieces. After washing with deionized water, the gel pieces were transferred into the microcentrifuge tubes (2 mL), destained in 1 mM DTT and incubated in elution buffer (5 mM DTT, 50 mM Tris HCl, pH 7,9, 0,1% SDS, 0,15 M NaCl, and 0,1 mM EDTA), overnight, on a rotary shaker, at room temperature. The gel pieces are removed from the solution after centrifugation (12000 rpm, 15 min). The detergents from the samples are removed by adding ice cold acetone and incubating over night at −20°C. Pellets obtained after centrifugation 15 min at 12000 rpm were dissolved in 1,5 M Tris HCl pH 8,8 containing 2% SDS. Protein concentration was determined using DC protein assay kit (BioRad). 

### 2.7. Enzyme Linked Immunosorbent Assay (ELISA)

Sera from patients were tested on their reactivity with gangliosides (GM1, AG1) and with glycoproteins purified from human peripheral nerve and from both *Cj* O:19 isolates. Wells of the 96-well plate (flat bottom, high binding, Corning, NY, USA) were coated overnight at 4°C, with antigen (0.2 *μ*g/mL gangliosides, 4 *μ*g/mL protein) and with BSA (10 *μ*g/mL). Wells were blocked with 1% BSA/PBS, 2 h at room temperature, and washed four times with washing buffer (0.9% NaCl, 0.05% Tween 20, and 0.02% sodium azide). Sera from patients were added to the wells, in duplicate, at serial dilutions (1 : 100, 1 : 200, 1 : 400, 1 : 800, 1 : 1600, 1 : 3200, 1 : 6400, and 1 : 12800) in 0.1% BSA and 0.1% Tween 20/PBS and incubated over night at room temperature. Following washing, anti human IgG and anti human IgM, conjugated with peroxidase (Sigma-Aldrich), diluted 1 : 1000, were added as secondary antibody. After washing the plates, peroxidase substrate (OPD tablets, Sigma-Aldrich) was added to the wells, and the optical densities of the developed color were measured spectrophotometrically at 450 nm on ELISA reader (VICTOR X3, Perkin Elmer, USA). Average of the obtained values was calculated for each dilution and corrected by subtracting with the blank. Sera with the value of the OD greater twice than the negative control were taken as positive. The titer was defined as the highest sera dilution which gives positive reactivity. 

## 3. Results

### 3.1. Antiganglioside Antibodies in Tested Patients

Results from ELISA testing (Table 1) of the reactivity of patient sera with human gangliosides (GM1 and AG1) showed presence of anti-GM1 IgM antibodies in sera from 11 patients with MMN (45.83%). Presence of anti GM1 IgG antibodies was detected in only one (6.5%) of all of the tested patients with MMN, while IgG positive reactivity to AG1 was found in 2 patients (9.3%). Sera from patients with other neurological diseases did not show reactivity to either ganglioside. From the healthy controls, only one (4%) showed positive reactivity to GM1 ganglioside.

### 3.2. Western Blot

**Figure 1:**
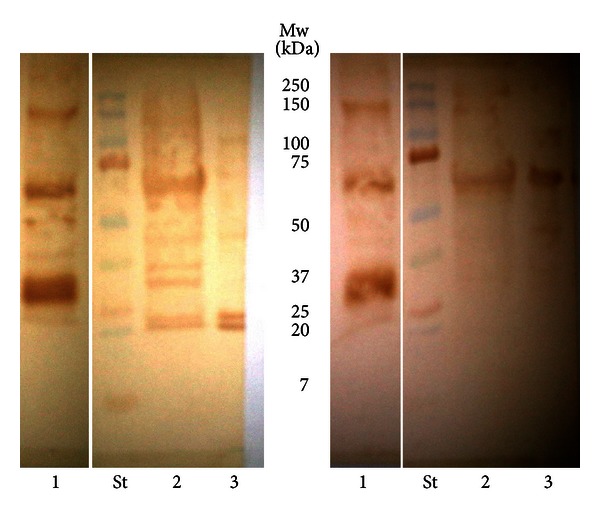
Western blot analysis of the isolated proteins: (1) human peripheral nerve, (2) *C. jejuni* O:19-GBS, and (3) *C. jejuni* O:19-enteritis, incubated with sera from patients with MMN.

**Figure 2:**
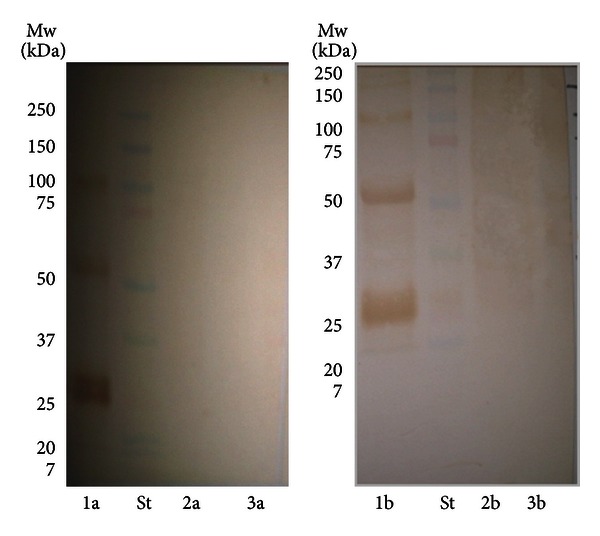
Western blot analysis of the isolated proteins: (1) human peripheral nerve, (2) *C. jejuni* O:19-GBS, and (3) *C. jejuni* O:19-enteritis, incubated with serum from patient with (a) MND and (b) MSMN.

Western blots showed positive reactivity of total 6 patients (25%) with MMN to several positive reactive bands present in the protein isolate from human peripheral nerve and from both *Cj* O:19 isolates (Table 2). All of the 6 sera with positive reactivity to isolated proteins were also positive on anti-GM1 IgM antibodies. Positive reactive bands with similar electrophoretic mobility in all of the three isolates were detected (60–62 kDa, 48–51 kDa, 42 kDa, and 38 kDa) in sera from three patients (12.5%) with MMN. All six patients showed positive reactivity to 48–52 kDa protein isolated from human peripheral nerve. From patients with other neurological diseases, 2 patients (40%) showed positive reactivity to the same antigen, but there was no reactivity to other proteins from any isolate. One of the negative controls (4%) showed positive reactivity to 60–62 kDa protein present in the three isolates and to 48–50 kDa isolated from human peripheral nerve. Representative blots are shown in Figures 1 and 2.

### 3.3. ELISA

Sera from the six patients that showed positive reactivity on western blot were tested on ELISA in order to determine the titer of antibodies to the 60–62 kDa and 48–51 kDa glycoproteins (Table 3). Increased titer (1 : 3200–1 : 12800) of IgG antibodies to 60–62 kDa protein isolated from human peripheral nerve was determined in sera from 3 patients with MMN. Positive reactivity and increased titer (1 : 400–1 : 12800) of IgG antibodies to 60–62 kDa protein isolated from *Cj* O:19-GBS associated were confirmed in sera from the same three patients that showed also positive reactivity to peripheral nerve antigen with the same electrophoretic mobility. One of these patients had a previous history of *Cj* infection. Only one of the tested sera showed positive IgG reactivity to the 60–62 kDa antigen isolated from *Cj* O:19-enteritis associated.

Positive IgG reactivity to 48–51 kDa protein isolated from human peripheral nerve showed all of the tested sera (titer 1 : 200–1 : 800), while only one sera showed positive reactivity (titer 1 : 200), to the antigen with similar electrophoretic mobility isolated from *Cj* O:19-GBS associated. None of the 6 tested sera showed positive reactivity to 48–51 kDa protein isolated from *Cj* O:19-enteritis associated.

## 4. Discussion

In 11 out of 24 patients with MMN (45.8%) included in the study serum IgM antibodies to GM1 have been detected. Previous series of patients with MMN have rates of positive GM1 antibodies ranging from 25 to 80% [19–21]. A consensus statement of the AAEM included GM1 antibody in the supportive laboratory criteria for MMN [22]. The sera from patients with MMN showed cross-reactivity to proteins isolated from human peripheral nerve and from *Cj* O:19. Four positive reactive proteins were obtained in (60–62 kDa, 48–51 kDa, 42 kDa, and 38 kDa) in all of the three isolates. The cross-reactivity of these proteins to PNA indicate on the glycoprotein structure and presence of the GalGalNAc determinant [17]. The correlation between the presence of IgM anti-GM1 antibodies and antibodies to 48–51 kDa PNA-binding glycoprotein from human peripheral nerve in patients with MMN could not be concluded from this study, since sera from patients with other neuropathies showed also positive reactivity to these bands. The study should be performed on more patients with MMN and controls. There is also cross-reactivity of the sera from two patients with MMN- and PNA-binding glycoproteins with mobility between 60–62 kDa and 48–51 kDa isolated from *Cj. *Sera from patients with other neuropathies did not show any reactivity to the tested antigens, except with the 48–51 kDa protein isolated from human peripheral nerve. Western blot results have shown that there is no significant difference in the reactivity of tested sera with the proteins isolated from *Cj*-GBS associated or *Cj*-enteritis associated. In contrast the ELISA results have shown significant difference in the reactivity of sera to antigens from different bacterial isolates. Three patients with MMN showed IgG antibodies to 60–62 kDa   glycoproteins isolated from *Cj *O:19 GBS and positive IgG cross-reactive antibodies to peripheral nerve antigen with the same electrophoretic mobility. One of these patients had an increased titer of IgM anti-GM1 antibodies and previous history of *Cj* infection which suggests a possible mechanism of molecular mimicry in the pathogenesis of MMN [9, 11]. The mechanism of generation of both IgG antibodies to glycoproteins and IgM antibodies to gangliosides has to be further investigated. There is a higher reactivity of tested sera to glycoproteins isolated from *Cj-*GBS associated, compared to proteins isolated from *Cj*-enteritis associated. Variability of the response obtained with both methods could be a result of conformational differences, caused by different interaction of the antigen with different matrices. There is also a possibility that different reactivity of sera is due to the different specificity of antibodies used in the immunological reaction [8]. The possibility that there is a different conformational determinant present in the proteins isolated from *Cj*-GBS associated, shared by the proteins from human peripheral nerve, which is recognized by the antibodies in sera from patients with MMN, is yet to be elucidated. 

The cross-reactivity of antibodies from tested sera to human peripheral nerve antigens and antigens isolated from *Cj* could be explained by the concept of natural autoantibodies, reflecting the natural nonpathogenic autoimmunity or by the presence of antibodies generated from infections and natural exposure to pathogens, including *Cj* [23–25]. This explains the positive reactivity of the one serum from the healthy controls to 60 kDa glycoproteins.

The increased titer of IgG antibodies to human peripheral nerve antigens in patients with MMN could be a secondary reaction to the exposure of nonimmunogenic nerve antigens, following demyelination and nerve cell damage [25]. These autoantibodies may further contribute to the progression and deterioration of the disease. 

The higher frequency of the presence of antiganglioside and antiglycoprotein antigens in patients with MMN compared to healthy controls and to patients with other neuropathies indicates on their possible pathogenic significance in the inducing and propagation of the nerve damage and development of neurological symptoms. The cross-reactivity of these antibodies to *Cj* antigens indicates on the shared epitopes between human and bacterial glycoproteins and their possible role in the induction of autoantibodies and peripheral neuropathies following infection with *Campylobacter jejuni*. Determination of the structure and localization of the cross-reactive PNA-binding glycoproteins will help in understanding the mechanisms that trigger myelin-related neurological diseases, including MMN. 

## Figures and Tables

**Table 1:** ELISA reactivity of tested patient sera with gangliosides.

Diagnose	MMN (*n* = 24)	OND (*n* = 5)	HC (*n* = 24)
Anti-GM1 IgM	11 (45.83%)	0	1 (4%)
Anti-GM1 IgG	1 (6.5%)	0	0
Anti-AG1 IgM	0	0	0
Anti-AG1 IgG	2 (9.3%)	0	0

MMN: multifocal motor neuropathy; OND: other neurological diseases; HC: healthy controls.

**Table 2:** Results from Western blot: number of patients with positive reactivity to isolated proteins.

Patients	Antigen											
	60–62 kDa protein			48–51 kDa protein			42 kDa protein			38 kDa protein		
	PN	*Cj*GBS	*Cj-*ent.	PN	*Cj*GBS	*Cj-*ent.	PN	*Cj*GBS	*Cj-*ent.	PN	*Cj*GBS	*Cj-*ent.
MMN (*n* = 6)	3	3	3	6	3	3	3	3	3	3	3	3
ON (*n* = 2)	0	0	0	2	0	0	0	0	0	0	0	0
HC (*n* = 24)	1	1	1	1	0	0	0	0	0	0	0	0

MMN: multifocal motor neuropathy; OND: other neurological diseases; HC: healthy controls, PN: human peripheral nerve; *Cj*GBS: *Campylobacter jejuni*-GBS associated; *Cj*-ent.: *Campylobacter jejuni*-enteritis associated.

**Table 3:** Titer of IgG antibodies to purified glycoproteins from human peripheral nerve, *Cj* O:19-GBS, and *Cj* O:19-enteritis.

Patients	Diagnose	Antigen					
		60–62 kDa protein			48–51 kDa protein		
		PN	*Cj*GBS	*Cj-*ent.	PN	*Cj*GBS	*Cj-*ent.
1	MMN	1 : 12800	1 : 12800	—	1 : 800	1 : 200	—
2	MMN	1 : 3200	1 : 400	1 : 400	1 : 200	—	—
3	MMN	1 : 12800	1 : 800	—	1 : 800	—	—

MMN: multifocal motor neuropathy; PN: human peripheral nerve, *Cj*GBS: *Campylobacter jejuni*-GBS associated; *Cj*-ent.: *Campylobacter jejuni*-enteritis associated.

## References

[1] Nobile-Orazio E (2001). Multifocal motor neuropathy. *Journal of Neuroimmunology*.

[2] Ghosh A, Busby M, Kennett R, Mills K, Donaghy M (2005). A practical definition of conduction block in IvIg responsive multifocal motor neuropathy. *Journal of Neurology, Neurosurgery and Psychiatry*.

[3] Willison HJ, Veitch J, Swan AV (1999). Inter-laboratory validation of an ELISA for the determination of serum anti-ganglioside antibodies. *European Journal of Neurology*.

[4] Yuki N, Yoshino H, Sato S, Miyatake T (1990). Acute axonal polyneuropathy associated with anti-GM1 antibodies following *Campylobacter* enteritis. *Neurology*.

[5] Cats EA, Jacobs BC, Yuki N (2010). Multifocal motor neuropathy: association of anti-GM1 IgM antibodies with clinical features. *Neurology*.

[6] Ho TW, Willison HJ, Nachamkin I (1999). Anti-GD1a antibody is associated with axonal but not demyelinating forms of Guillain-Barré syndrome. *Annals of Neurology*.

[7] Yuki N, Ho TW, Tagawa Y (1999). Autoantibodies to GM1b and GalNAc-GD1a: relationship to *Campylobacter jejuni* infection and acute motor axonal neuropathy in China. *Journal of the Neurological Sciences*.

[8] Wirguin I, Suturkova-Milosevic L, Della-Latta P, Fisher T, Brown RH, Latov N (1994). Monoclonal IgM antibodies to GM1 and asialo-GM1 in chronic neuropathies cross-react with *Campylobacter jejuni* lipopolysaccharides. *Annals of Neurology*.

[9] White JR, Sachs GM, Gilchrist JM (1996). Multifocal motor neuropathy with conduction block and *Campylobacter jejuni*. *Neurology*.

[10] Abbruzzese M, Reni L, Schenone A (1997). Multifocal motor neuropathy with conduction block after *Campylobacter jejuni* enteritis. *Neurology*.

[11] Taylor BV, Phillips BA, Speed BR, Kaldor J, Carroll WM, Mastaglia FL (1998). Serological evidence for infection with *Campylobacter jejuni*lcoli in patients with multifocal motor neuropathy. *Journal of Clinical Neuroscience*.

[12] Terenghi F, Allaria S, Scarlato G, Nobile-Orazio E (2002). Multifocal motor neuropathy and *Campylobacter jejuni* reactivity. *Neurology*.

[13] Walsh FS, Cronin M, Koblar S (1991). Association between glycoconjugate antibodies and campylobacter infection in patients with Guillain-Barre syndrome. *Journal of Neuroimmunology*.

[14] Fujimoto S, Yuki N, Itoh T, Amako K (1992). Specific serotype of *Campylobacter jejuni* associated with Guillain-Barre syndrome. *Journal of Infectious Diseases*.

[15] Brezovska K, Poceva Panovska A, Grozdanova A, Suturkova Lj, Basta I, Apostolski S (2011). Immunoreactivity of glycoproteins isolated from human peripheral nerve and *Campylobacter jejuni* (O:19). *Journal of Neurosciences in Rural Practice*.

[16] Poceva-Panovska A, Brezovska K, Grozdanova A, Apostolski S, Suturkova Lj (2011). Immunoreactivity and characterization of oligosaccharide determinants in glycoproteins isolated from peripheral nerve and *Campylobacter jejuni* O:19. *Neurologia Croatica*.

[17] Apostolski S, Sadiq SA, Hays A (1994). Identification of Gal(*β*1-3)GalNAc bearing glycoproteins at the nodes of Ranvier in peripheral nerve. *Journal of Neuroscience Research*.

[18] Liu X, McNally DJ, Nothaft H, Szymanski CM, Brisson JR, Li J (2006). Mass spectrometry-based glycomics strategy for exploring N-linked glycosylation in eukaryotes and bacteria. *Analytical Chemistry*.

[19] Slee M, Selvan A, Donaghy M (2007). Multifocal motor neuropathy: the diagnostic spectrum and response to treatment. *Neurology*.

[20] Adams D, Kuntzer T, Burger D (1991). Predictive value of anti-GM1 ganglioside antibodies in neuromuscular diseases: a study of 180 sera. *Journal of Neuroimmunology*.

[21] Kornberg AJ, Pestronk A (1994). The clinical and diagnostic role of anti-GM1 antibody testing. *Muscle and Nerve*.

[22] Olney RK, Lewis RA, Putnam TD, Campellone JV (2003). Consensus criteria for the diagnosis of multifocal motor neuropathy. *Muscle and Nerve*.

[23] Avrameas S (1991). Natural autoantibodies: from “horror autotoxicus” to “gnothi seauton”. *Immunology Today*.

[24] Panigrahi P, Losonsky G, DeTolla LJ, Morris JG (1992). Human immune response to *Campylobacter jejuni* proteins expressed in vivo. *Infection and Immunity*.

[25] Gabriel CM, Gregson NA, Hughes RAC (2000). Anti-PMP22 antibodies in patients with inflammatory neuropathy. *Journal of Neuroimmunology*.

